# Agreement and Classification Performance of a Mobile Self-Triage Application Based on the Manchester Triage System in Hemodynamically Stable Adult Walk-In Emergency Department Patients: A Prospective Observational Study

**DOI:** 10.3390/jcm15176702

**Published:** 2026-08-29

**Authors:** Ahmet Buğra Önler, Suha Serin, Bahadir Caglar

**Affiliations:** 1 Department of Emergency Medicine, Balikesir State Hospital, 10020 Balikesir, Turkey; bugraonler@gmail.com; 2 Department of Emergency Medicine, Faculty of Medicine, Balikesir University, 10145 Balikesir, Turkey; suhaserin@gmail.com

**Keywords:** emergency department, Manchester Triage System, self-triage, mobile health, digital triage, triage accuracy, overtriage, undertriage, patient self-assessment, emergency department crowding

## Abstract

**Background/Objectives:** Digital self-triage tools may support patient-flow management in crowded emergency departments (EDs), but their agreement with established professional triage systems requires prospective validation. We evaluated the agreement and classification performance of a locally developed mobile self-triage application, structured according to Manchester Triage System (MTS)-related urgency logic, compared with professional MTS assessment in adult ED patients. **Methods:** This prospective, observational, single-center agreement study included 2274 consecutive, hemodynamically stable adult walk-in patients who used a tablet-based application covering 11 predefined presenting complaints and subsequently underwent a single, blinded professional MTS assessment, which served as the prespecified comparator. Of 3244 initial registrations, 970 (29.9%) were excluded because their complaints were not covered by the predefined pathways. MTS Categories 2–3 were classified as urgent. **Results:** Exact agreement occurred in 968 patients (42.6%); 1161 patients (51.1%) were overtriaged and 145 (6.3%) were undertriaged. The application showed 93.9% sensitivity, 28.7% specificity, 61.1% positive predictive value, and 79.8% negative predictive value for identifying professional MTS-defined urgent cases. Agreement was slight by unweighted kappa (κ = 0.198), fair by quadratically weighted kappa (κw = 0.278), and the prevalence- and bias-adjusted kappa for binary urgent/non-urgent classification was 0.28. Overtriage exceeded 70% for headache and low back pain, and these complaints were the strongest independent predictors of overtriage; higher self-reported pain intensity was strongly associated with overtriage. **Conclusions:** The application demonstrated a conservative classification profile with high sensitivity and a low undertriage rate but frequent overtriage that would inflate the apparent urgent caseload if the tool were used unsupervised; it should be considered an adjunct to, rather than a replacement for, professional triage. Findings apply to stable adult walk-in patients whose complaints matched the predefined categories and may not generalize to the broader ED population.

## 1. Introduction

Emergency departments (EDs) provide uninterrupted 24 h access to acute care and represent a critical interface between patients and the healthcare system [1,2]. ED attendance has increased worldwide. In the United States, more than 155 million ED visits were recorded in 2022 [3]. A similar pattern is evident in Türkiye, where the 2023 Health Statistics Yearbook reported more than 150 million ED visits in a population exceeding 85 million, corresponding to approximately 175–180 visits per 100 inhabitants [4]. In the International Federation for Emergency Medicine 2022 survey of 41 countries, all participating countries reported ED crowding [5].

ED crowding results from an imbalance between patient demand and available resources. It is associated with prolonged waiting times, delayed diagnosis and treatment, lower patient satisfaction, increased morbidity and mortality, and burnout among healthcare professionals [6,7]. More than one-third of ED visits in some settings involve non-urgent conditions that could potentially be managed in primary care [8,9]. In this context, triage—the prioritization of patients according to clinical urgency—is essential for safe and efficient ED operation [10].

International evidence indicates that five-level triage systems have better reliability, discrimination, validity, and inter-rater agreement than two- or three-level systems [11,12]. The Manchester Triage System (MTS) is an evidence-based five-level triage system used widely, particularly in European EDs. It assigns patients to categories ranging from Category 1 (immediate) to Category 5 (non-urgent) using 55 symptom-specific flowcharts [13]. The MTS has demonstrated moderate-to-good validity across patient populations and high sensitivity for high-acuity presentations [14].

Increasing patient volumes and insufficient staffing can compromise conventional triage performed by trained nurses. Heavy workloads may prolong pre-triage waiting, lead to incomplete urgency assessments, and increase triage discordance [15]. These pressures have stimulated interest in digital self-triage systems that allow patients to report symptoms before or immediately after arrival using algorithmic decision support. Kiosk- and application-based self-triage systems, evaluated mainly in North America and Europe, may shorten waiting times, facilitate earlier recognition of high-acuity patients, and reduce non-urgent ED attendance [16,17].

Systematic reviews of digital symptom assessors have reported wide variation in triage agreement, ranging from approximately 33% to 90% across platforms and settings [18,19]. A consistent finding is that these tools tend to overtriage, largely because of risk-averse design. Although such conservatism may protect patient safety, it may also reduce operational efficiency. Importantly, many evaluated platforms are commercial products with non-standardized algorithms and have not been prospectively validated against established professional triage systems in real-world ED settings [20].

Few studies have compared mobile self-triage directly with professional MTS triage. Dickson et al. compared an electronic self-triage tool with nurse-administered MTS in two UK EDs and reported overtriage and undertriage rates of 59.2% and 10.1%, respectively [21]. Trivedi et al. found 27% agreement between computer-assisted self-triage and nurse triage in Canada [22]. Gilbert et al. evaluated the Belgian ODISSEE platform in a scenario-based design and reported variable agreement across diagnostic categories [23]. However, prospective evaluation of a locally developed tablet application structured according to selected MTS-related urgency logic in a real-time ED environment, particularly in a middle-income country with exceptionally high ED utilization, remains limited.

We therefore developed a mobile self-triage application using Android Studio and Kotlin. The application incorporated decision algorithms structured according to selected MTS-related urgency logic for 11 common presenting-complaint categories. The primary objective of this study was to assess agreement between the application and professional MTS triage in the ED. Secondary objectives were to evaluate the application’s performance in identifying MTS-defined urgent patients and to identify patient- and symptom-specific predictors of triage discordance.

## 2. Materials and Methods

### 2.1. Study Design and Ethics

This prospective, observational, single-center agreement study was conducted in the ED of Balıkesir University Health Practice and Research Hospital, a tertiary care center with approximately 200–250 adult ED visits per day. The study was conducted in accordance with the Declaration of Helsinki and approved by the Balıkesir University Non-Interventional Health Sciences Research Ethics Committee (Decision No. 2025/257; 1 July 2025). Written informed consent was obtained from all participants before enrollment. The study is reported in accordance with the principles of the STROBE statement for observational studies.

### 2.2. Participants

Adults aged 18 years or older who presented to the ED as walk-in patients between 1 July and 15 October 2025, had stable vital signs (systolic blood pressure >90 mmHg, heart rate 60–100 beats/min, respiratory rate 12–20 breaths/min, oxygen saturation >92%, and Glasgow Coma Scale score of 15), and were assigned to the yellow or green category by the triage nurse according to the institutional three-level triage system were assessed for eligibility. Consecutive sampling was used. Hemodynamic stability was an eligibility criterion rather than a proxy for low clinical urgency. In the MTS, urgency categories are assigned on the basis of symptom-specific discriminators—such as severe pain, sudden or recent onset, persistent vomiting, or high-risk historical features—rather than vital-sign derangement alone [13]. Accordingly, patients with entirely stable vital signs may still meet Category 2 (very urgent) or Category 3 (urgent) discriminators, and such ambulatory presentations constituted the clinically relevant target population for a patient-facing self-triage tool. Patients were excluded for any of the following reasons: red triage category or unstable vital signs; arrival by ambulance; trauma; inability to use the tablet application independently, including unaccompanied visual or hearing impairment or inability to speak Turkish; age younger than 18 years; or refusal to participate. Patients whose complaints did not match one of the available algorithms selected “Other” and were excluded from the analytical cohort.

### 2.3. Mobile Self-Triage Application

The application was developed by the study team using Kotlin in the Android Studio development environment (Google LLC, Mountain View, CA, USA) [24,25]. The application ran on a Samsung Galaxy Tab S7 tablet (Samsung Electronics Co., Ltd., Suwon, Republic of Korea). A native Android tablet application was chosen for several practical reasons. Android tablets are low-cost and widely available, which is important for scalability in a high-volume public hospital; the platform supports kiosk-style deployment in which the device is locked to a single full-screen application with internet access disabled, meeting the data-security requirements of the study; and native development in Kotlin enabled fully offline operation with local data storage, automatic reset between patients, and straightforward implementation of the touch-based questionnaire and pain-intensity slider. The application was purpose-built by the study team for this study rather than adapted from a commercial product, ensuring that its decision logic corresponded exactly to the MTS-aligned algorithms described below. Participants used the application on a designated tablet. They first entered basic demographic information and then selected one of 11 predefined complaint categories: abdominal pain, sore throat, headache, low back pain, diarrhea and vomiting, chest pain, urinary tract problems, dizziness, eye problems, allergic symptoms, and shortness of breath.

Complaint-specific decision algorithms were developed by a multidisciplinary team of emergency medicine faculty members and residents with reference to the urgency hierarchy and symptom-specific discriminator logic of the Manchester Triage System [13]. The application was developed as a research prototype and did not reproduce the full MTS flowcharts in the manuscript or user interface. The complete question sets, decision boxes, and category assignment rules for all 11 complaint pathways are provided in Supplementary Material S3. During the study, the application output was not used to determine clinical care and did not replace standard professional triage. Each algorithm used a three-stage sequential Yes/No questioning structure: stage 1 evaluated very urgent features corresponding to Category 2; stage 2 evaluated urgent features corresponding to Category 3; and stage 3 distinguished lower-acuity Categories 4 and 5. For pain-related complaints (headache, low back pain, sore throat, chest pain, and abdominal pain), a 0–10 numerical pain-intensity slider reflecting the MTS pain scale was integrated into the decision pathway. The pain-intensity slider was positioned as the terminal node of these pathways: it was displayed only when the patient had not endorsed any higher-acuity discriminator in the preceding decision boxes, in which case the pain score alone determined the category (8–10, Category 2; 5–7, Category 3; 1–4, Category 4; 0, Category 5). Patients who endorsed a Category 2 or 3 discriminator were assigned to that category and did not reach the slider. The slider was initially displayed at the midpoint (5) and could be confirmed without being moved. The self-triage category generated by the application (Categories 2–5) was displayed on the screen and stored in the tablet’s local database. Internet access was disabled for data security, and the application was automatically reset after each patient.

To reduce user burden, the interface used short questions, binary response options, and complaint-specific pathways. No free-text symptom interpretation was performed by the application. All triage outputs were generated through deterministic rule-based algorithms rather than machine-learning prediction.

### 2.4. Development of MTS-Aligned Self-Triage Algorithms

The complaint-specific self-triage algorithms were developed through a structured clinical mapping process. First, the study team identified 11 common presenting complaints that were considered suitable for patient-facing digital self-triage and frequently encountered in the study ED. Complaint categories were selected by consensus of the emergency medicine study team according to three criteria: (i) high presentation frequency among ambulatory walk-in patients in the study ED; (ii) availability of a corresponding MTS flowchart whose discriminators could be reformulated as plain-language questions answerable by lay users without physical examination, vital-sign measurement, or clinician interpretation; and (iii) suitability for a binary Yes/No questioning format. Complaints that primarily require objective assessment, such as trauma-related presentations, or that are difficult for patients to self-characterize were not included in this prototype. For each complaint category, the corresponding Manchester Triage System flowchart and discriminators were reviewed by emergency medicine faculty members and residents. Clinician-facing MTS discriminators were then converted into plain-language, patient-facing Yes/No questions while preserving the original urgency hierarchy of the MTS framework.

Each algorithm followed a sequential rule-based structure. Questions corresponding to higher-acuity discriminators were presented first. A positive response to a Category 2 discriminator assigned the patient to Category 2. If no Category 2 feature was present, the application proceeded to Category 3 discriminators. If no Category 3 feature was present, additional questions were used to distinguish Category 4 from Category 5. For pain-related complaints, a 0–10 numerical rating scale was incorporated, and pain severity was mapped to the relevant MTS pain discriminator. The final self-triage category was determined automatically by the highest-acuity positive discriminator identified during the sequence.

Before implementation, the algorithms and question wording were reviewed by the emergency medicine study team for clinical consistency, clarity, and appropriateness for lay users. The application interface was designed to minimize free-text entry and to allow completion using predefined responses. The application did not provide diagnostic advice or treatment recommendations; it only generated a triage-priority category. The output was not used to determine patient care during the study.

### 2.5. Professional MTS Assessment

After completing the self-triage application, all participants underwent independent professional triage assessment using the Manchester Triage System. Assessments were performed by emergency medicine specialists or emergency medicine residents working in the study ED. All assessors had prior clinical experience with structured ED triage and received a standardized briefing on the study protocol and MTS-based categorization before patient enrollment. The assessors used the relevant MTS flowcharts and discriminators to assign a professional MTS category between 2 and 5.

Clinicians were blinded to the self-triage output at the time of assessment. The professional MTS category was recorded in the patient file and subsequently matched with the application output for analysis. For the purposes of this study, professional MTS assessment was used as the prespecified comparator for agreement and classification-performance analyses because it represented the structured clinical triage decision made by trained emergency clinicians using the same MTS framework with which the application algorithms were aligned. Because no second independent assessment or adjudication panel was used, this comparator should not be interpreted as an error-free clinical gold standard.

### 2.6. Outcomes and Definitions

The primary outcome was exact agreement, defined as identical self-triage and professional MTS categories. Overtriage was defined as assignment by the application to a higher-acuity category than professional MTS (i.e., a lower MTS category number). Undertriage was defined as assignment by the application to a lower-acuity category than professional MTS (i.e., a higher MTS category number). For binary performance analyses, MTS Categories 2 and 3 were classified as urgent and Categories 4 and 5 as non-urgent, representing the clinically relevant threshold for prioritization.

### 2.7. Statistical Analysis

Statistical analyses were performed using IBM SPSS Statistics version 25.0 (IBM Corp., Armonk, NY, USA). Normality of continuous variables was assessed visually using histograms and analytically using the Kolmogorov–Smirnov test. Categorical variables are presented as counts and percentages, and continuous variables as mean ± standard deviation (SD). Categorical groups were compared using the chi-square test or Fisher’s exact test, as appropriate. Continuous variables were compared between two groups using the independent-samples *t* test and among three groups using one-way analysis of variance (ANOVA). When the overall ANOVA was significant, pairwise comparisons were performed using the Bonferroni post hoc test. Agreement between self-triage and professional MTS categories was assessed using unweighted Cohen’s kappa and quadratically weighted kappa, interpreted according to the criteria of Landis and Koch [26]. Performance in identifying MTS-defined urgent patients was evaluated by calculating sensitivity, specificity, positive predictive value (PPV), negative predictive value (NPV), and overall accuracy. Separate binary logistic regression models were constructed for overtriage and undertriage. Age, sex, educational level, time of presentation, and complaint category were included in the models. Model discrimination was assessed using the area under the receiver operating characteristic curve (AUC). For classification performance measures, 95% confidence intervals (CIs) were calculated using the exact binomial method. Confidence intervals for kappa statistics were calculated using asymptotic standard errors. No a priori sample size calculation was performed; all eligible patients presenting during the study period were enrolled consecutively, and the resulting sample of 2274 patients yielded a 95% CI half-width of approximately ±2.0 percentage points for the primary agreement estimate. Statistical significance was set at *p* < 0.05. Model calibration was assessed with the Hosmer–Lemeshow test, explained variation with Nagelkerke’s R^2^, and multicollinearity with variance inflation factors. Complaint-specific sensitivity, specificity, and positive and negative predictive values were calculated with Wilson 95% confidence intervals, and overall positive and negative likelihood ratios were reported. Because Cohen’s kappa is sensitive to prevalence and marginal imbalance, the prevalence- and bias-adjusted kappa (PABAK) was reported in addition to conventional kappa statistics. The association between pain score and overtriage was additionally examined in a logistic model adjusted for complaint category, age, and sex. Patients excluded because they selected “Other” were compared with the analytical cohort with respect to age, sex, and educational level, and their discharge ICD-10 diagnoses, when available, were summarized by chapter to characterize the complaints not covered by the application.

## 3. Results

### 3.1. Study Population

**Figure 1:**
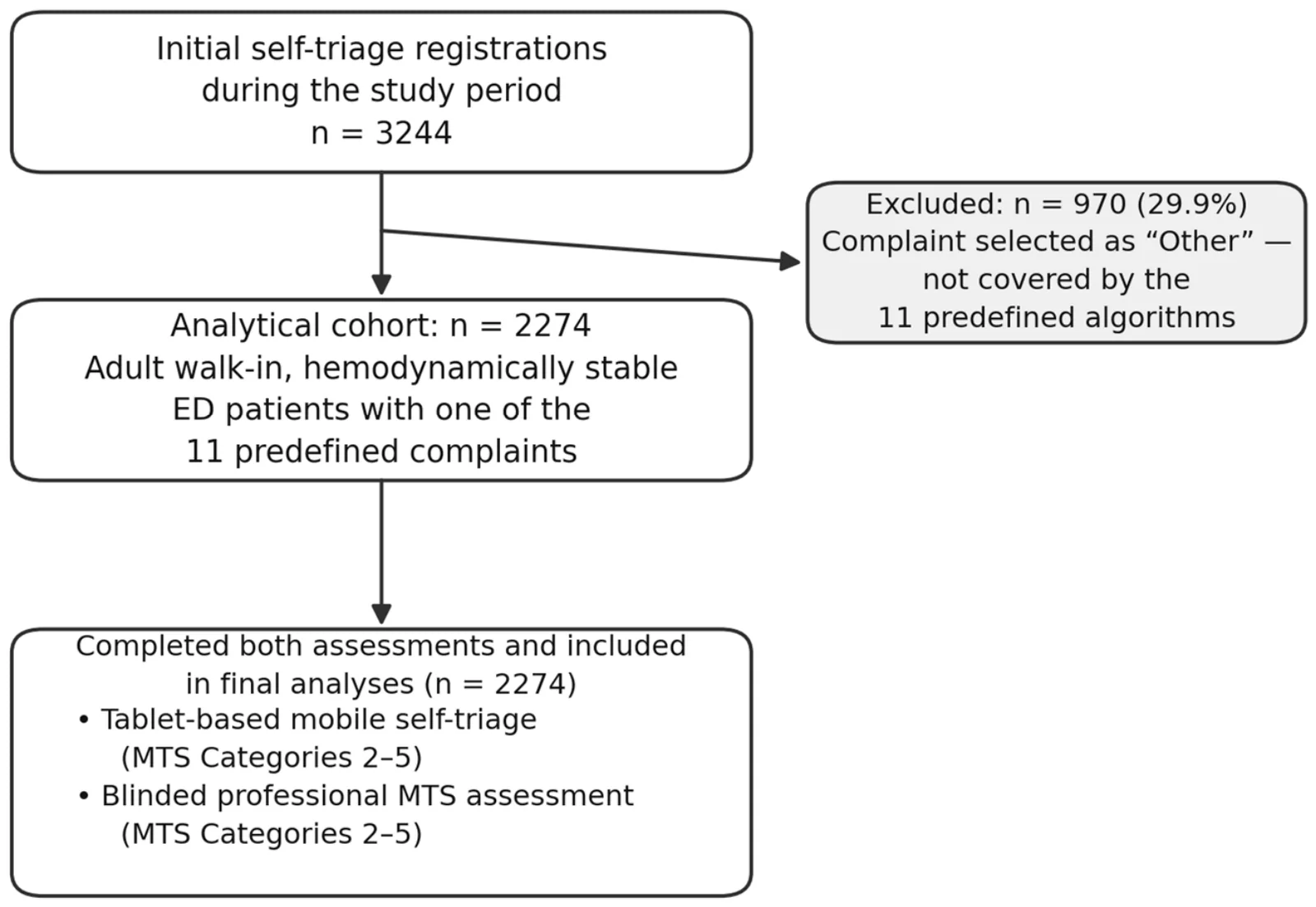
Participant flow diagram. MTS: Manchester Triage System.

During the study period, 3244 initial self-triage registrations were recorded. Of these, 970 patients (29.9%) selected “Other” because their presenting complaint could not be matched to one of the 11 predefined complaint categories available in the application and were excluded from the analytical cohort. Therefore, 2274 patients were included in the final analysis (Figure 1). Compared with the analytical cohort, patients who selected “Other” were slightly older (47.1 ± 18.7 vs. 44.4 ± 18.2 years; *p* < 0.001) but did not differ in sex distribution (53.3% vs. 55.0% female; *p* = 0.40) or educational level (*p* = 0.18) (Supplementary Table S1). A professional MTS category was recorded for only 57 of these patients (5.9%), precluding agreement analysis in this group. Discharge ICD-10 codes were available for 943 of the 970 excluded patients (97.2%); the most frequent chapters were diseases of the musculoskeletal system and connective tissue (33.9%, almost entirely soft-tissue and limb-pain codes), followed by circulatory (8.8%), symptom-based (R codes; 7.0%), digestive (6.6%), and respiratory (6.5%) diagnoses (Supplementary Table S2). This distribution indicates that the “Other” group was dominated by musculoskeletal and extremity complaints, which were not represented among the 11 predefined categories, rather than by patients unable to use the application.

The mean age was 44.4 ± 18.2 years (median, 42 years; interquartile range, 29 years; range, 18–94 years), and 1250 patients (55.0%) were female. The most common educational level was primary school (34.7%), followed by university (27.2%) and high school (24.1%). Most presentations occurred between 08:00 and 17:00 (57.8%). The most frequent presenting complaint was abdominal pain (19.6%), followed by sore throat (14.1%) and headache (12.0%). Patient characteristics are summarized in Table 1.

### 3.2. Distribution of Self-Triage and Professional MTS Categories

A marked difference was observed between the distributions of self-triage and professional MTS categories (Table 2). Self-triage assigned 43.3% of patients to Category 2, whereas professional MTS assigned 13.6% to this category. In contrast, professional MTS classified 43.4% of patients as Category 4, whereas self-triage classified 13.7% as Category 4. Overall, self-triage classified 1902 patients (83.6%) as urgent (Categories 2–3), compared with 1238 patients (54.4%) by professional MTS.

### 3.3. Triage Agreement

Exact agreement occurred in 968 patients (42.6%), whereas 1161 patients (51.1%) were overtriaged and 145 (6.3%) were undertriaged. The cross-classification matrix of self-triage and professional MTS categories is shown in Table 3. Most disagreements occurred between adjacent triage categories. Among all patients, a one-category difference was observed in 942 patients (41.4%), a two-category difference in 352 patients (15.5%), and a three-category difference in 12 patients (0.5%). Among discordant cases only, these represented 72.1%, 27.0%, and 0.9% of disagreements, respectively.

### 3.4. Classification Performance in Identifying Urgent Patients

Agreement between self-triage and professional MTS was slight according to unweighted Cohen’s kappa (κ = 0.198; 95% CI, 0.173–0.223; *p* < 0.001) and fair according to quadratically weighted kappa (κw = 0.278; 95% CI, 0.246–0.310; *p* < 0.001) [26]. Overall observed agreement for the binary urgent/non-urgent classification was 64.2%, corresponding to a PABAK of 0.28 (four-category PABAK, 0.23). The positive and negative likelihood ratios for urgent classification were 1.32 and 0.21, respectively. The higher weighted kappa indicates that most disagreements were confined to adjacent categories. In binary analysis using professional MTS as the comparator and Categories 2–3 as the urgent threshold, the application classified 1163 patients as true positives, 739 as false positives, 75 as false negatives, and 297 as true negatives. This corresponded to a sensitivity of 93.9% (95% CI, 92.5–95.2), specificity of 28.7% (95% CI, 25.9–31.5), PPV of 61.1% (95% CI, 58.9–63.3), NPV of 79.8% (95% CI, 75.4–83.8), and overall accuracy of 64.2% (95% CI, 62.2–66.2) (Table 4).

### 3.5. Factors Associated with Triage Discordance

Triage outcomes differed significantly across age groups (*p* < 0.001; Cramer’s V = 0.086). Overtriage was highest among patients aged 18–39 years (55.2%), compared with 50.0% among those aged 40–59 years and 42.3% among those aged 60 years or older. Undertriage was most frequent in the oldest group (11.5%). In multivariable analysis, each one-year increase in age was associated with a small increase in undertriage risk (adjusted odds ratio [aOR], 1.015; 95% CI, 1.004–1.028; *p* = 0.011) and a small decrease in overtriage risk (aOR, 0.987; 95% CI, 0.980–0.993; *p* < 0.001).

Sex was not associated with triage outcome (*p* = 0.953; Cramer’s V = 0.007), and educational level was not independently associated with discordance (*p* = 0.441). No statistically significant difference in triage agreement was observed across presentation-time intervals (*p* = 0.129; Cramer’s V = 0.040).

Presenting complaint was the strongest determinant of discordance (chi-square = 277.3, df = 20, *p* < 0.001; Cramer’s V = 0.247). Overtriage rates were highest for low back pain (73.0%) and headache (72.1%), followed by sore throat (62.0%) and allergic symptoms (61.1%). In contrast, urinary tract problems, diarrhea and vomiting, and eye problems had lower overtriage rates and higher concordance (Table 5). In multivariable regression with allergic symptoms as the reference category, headache (aOR, 1.69; 95% CI, 1.05–2.71; *p* = 0.030) and low back pain (aOR, 1.82; 95% CI, 1.11–2.97; *p* = 0.017) were the only complaints independently associated with higher odds of overtriage, whereas urinary tract problems (aOR, 0.23; 95% CI, 0.13–0.40), diarrhea and vomiting (aOR, 0.22; 95% CI, 0.13–0.36), and eye problems (aOR, 0.30; 95% CI, 0.17–0.53) (all *p* < 0.001) were associated with lower odds. Relative to allergic symptoms, headache (aOR, 0.22; 95% CI, 0.09–0.55), low back pain (aOR, 0.19; 95% CI, 0.07–0.52), sore throat (aOR, 0.25; 95% CI, 0.11–0.59), dizziness (aOR, 0.24; 95% CI, 0.08–0.69), chest pain (aOR, 0.32; 95% CI, 0.13–0.80), and abdominal pain (aOR, 0.43; 95% CI, 0.21–0.88) were associated with lower odds of undertriage. Both models showed acceptable calibration (Hosmer–Lemeshow *p* = 0.39 for overtriage and *p* = 0.19 for undertriage) and modest discrimination (AUC, 0.695; 95% CI, 0.674–0.717, and AUC, 0.702; 95% CI, 0.658–0.745, respectively; Nagelkerke R^2^ = 0.15 and 0.08); all variance inflation factors were below 5. Complaint-specific classification performance is shown in Table 6: sensitivity exceeded 90% for eight of the 11 complaints, but specificity was below 15% for headache, low back pain, chest pain, and dizziness, the complaints in which pain-intensity scoring or cautious high-acuity discriminators dominated the pathway. Regression results are summarized in Table 7. Eye problems showed the most balanced profile (sensitivity 81.1%, specificity 69.6%). Wide confidence intervals for specificity in chest pain, dizziness, and shortness of breath reflect the small number of MTS-non-urgent patients in these groups.

### 3.6. Pain Severity and Triage Discordance

Of the 1448 patients with pain-related complaints, 411 (28.4%) had a recorded pain-intensity score. Among the remaining 1037 patients, 840 were assigned to a triage category by an earlier higher-acuity discriminator and therefore, by design, did not reach the pain slider. For the remaining 197 patients, item-level pathway logs (individual question answers and slider values) were unavailable because of a temporary technical failure of the application’s local logging function during part of the study period, so whether they reached the pain slider could not be reconstructed. Overall, item-level logs were missing for 313 of 2274 patients (13.8%); the final self-triage category and all other study variables were recorded for these patients, so the primary analyses were unaffected.

Among 411 patients with pain-related complaints who reported a pain-severity score, scores were substantially higher in overtriaged patients (mean, 6.9 ± 1.8) than in concordantly triaged patients (4.6 ± 2.2) and undertriaged patients (2.4 ± 2.2; one-way ANOVA, *p* < 0.001). Pairwise comparisons were statistically significant among all three groups (Table 8). This finding identifies subjective pain severity as a major driver of overtriage and is consistent with direct integration of pain scores into the MTS-aligned algorithms.

Among the 411 patients with a recorded pain score, 108 (26.3%) reported a score of exactly 5, more than twice the frequency of any adjacent value, consistent with confirmation of the default slider position. Under the pathway rules, scores of 5–7 assign Category 3; among the 202 patients in this band, only 78 (38.6%) were classified as urgent by professional MTS, and among the 101 patients scoring 8–10, 43 (42.6%) were classified as non-urgent. In a logistic model adjusted for complaint category, age, and sex, each 1-point increase in pain score was associated with 2.2-fold higher odds of overtriage (aOR, 2.24; 95% CI, 1.89–2.66; *p* < 0.001).

## 4. Discussion

### 4.1. Principal Findings

In this prospective single-center agreement study, a locally developed MTS-based mobile self-triage application was evaluated in real-world ED practice. Among 2274 adult walk-in patients, exact agreement with professional MTS triage was 42.6%. The application showed high sensitivity (93.9%) and a low undertriage rate (6.3%), but specificity was low (28.7%) and overtriage was frequent (51.1%). These results indicate a conservative classification profile rather than a precise replacement for clinician-led triage. Presenting complaint was the strongest determinant of discordance, and age had a statistically significant but modest independent effect.

### 4.2. Comparison with Previous Studies

The observed exact agreement is consistent with the broad range reported for digital self-triage systems in previous reviews [18,19]. Dickson et al. reported 59.2% overtriage and 10.1% undertriage when comparing electronic self-triage with nurse-administered MTS in UK EDs, broadly comparable with our findings [21]. Lammila-Escalera et al. reported undertriage rates of approximately 8–10.1% in a systematic review of kiosk-based self-triage systems [27]. Trivedi et al. found only 27% agreement between computer-assisted self-triage and nurse triage, highlighting the importance of algorithm design, symptom coverage, and implementation setting [22]. The relatively higher agreement in the present study may reflect the alignment of the application algorithms with the MTS-related urgency hierarchy and discriminator logic, thereby bringing the patient-facing decision logic closer to the professional reference framework.

### 4.3. Implications for Triage Prioritization and Implementation

For a self-triage tool intended to support ED prioritization, undertriage is a particularly important classification concern. The low undertriage rate and high sensitivity observed in this study suggest that the application rarely assigned professional MTS-defined urgent patients to non-urgent categories. This profile is consistent with previous evidence showing that digital triage tools frequently err toward higher urgency recommendations [28,29]. Such conservatism is clinically understandable because the consequence of missing a high-acuity case is potentially greater than that of assigning unnecessarily high priority. Nevertheless, the low specificity observed here, which parallels findings from other comparative evaluations of self-triage tools [30], indicates that the application should not be used as an independent gatekeeping instrument. The low specificity is best understood as an expected consequence of the deliberately conservative, risk-averse design of the algorithms rather than as purely random misclassification. Because the algorithms resolve any affirmative response to a high-acuity screening question toward the more urgent category, false-positive urgent classifications accumulate among lower-acuity patients, and specificity falls even while sensitivity remains high. Two design features contributed disproportionately: the direct mapping of patient-reported pain intensity onto MTS pain discriminators, which allowed severe subjective pain alone to trigger an urgent classification, and the absence of objective clinical inputs available during professional assessment, such as directly measured vital signs and clinician-observed findings. The pain-score analysis identifies two concrete targets: the default midpoint position of the slider, which appears to have been confirmed unchanged by a substantial minority of patients and automatically maps to an urgent category, and the 5–7 threshold for Category 3, within which fewer than 40% of patients were professionally judged urgent. Requiring an active pain selection and revising this threshold would be expected to improve specificity in pain-dominant presentations, subject to prospective re-validation. More generally, specificity could plausibly be increased by recalibrating patient-facing wording and pain-intensity thresholds for the complaints with the highest overtriage rates, adding brief confirmatory follow-up questions before an urgent classification is finalized, incorporating objective parameters such as kiosk-measured vital signs, and, as larger datasets accumulate, applying data-driven optimization of question thresholds. Any such recalibration must be prospectively re-validated to ensure that specificity gains are not achieved at the cost of increased undertriage.

The magnitude of overtriage warrants explicit emphasis. More than half of all patients (51.1%) and more than 70% of patients with headache or low back pain were assigned to a higher urgency category by the application than by professional triage. In an ED that already operates under sustained crowding, systematic overtriage of this magnitude is not a benign error: if acted upon, it would inflate the apparent urgent caseload, dilute the prioritization of patients who are truly urgent, increase demands on physician time and observation space, and could paradoxically prolong waiting for lower-acuity patients. Overtriage was not randomly distributed but concentrated in pain-dominant, low-acuity presentations, and it was strongly driven by self-reported pain intensity, which points to specific and correctable design features rather than to an inherent limitation of patient self-assessment. Until such recalibration has been achieved and re-validated, the application output should be treated as advisory and should never bypass professional triage.

A related consideration concerns time-critical presentations. Acute stroke and acute coronary syndromes are managed through dedicated pathways in which pre-hospital notification, immediate professional recognition at the door, and strict treatment-time targets are critical. In acute ischemic stroke, delays in door-to-physician and imaging processes have been associated with prolonged door-to-needle time and reduced likelihood of timely thrombolysis [31]. Patients with such presentations were, by design, outside the scope of the present study: they typically arrive by ambulance or with an obvious neurological deficit and were excluded, and no dedicated stroke pathway was included among the 11 complaints. Nevertheless, some patients with time-critical conditions may self-select a generic complaint such as dizziness, headache, or chest pain. The application’s pathways for these complaints contain high-acuity discriminators (for example, new focal weakness or speech disturbance in the dizziness and headache pathways, and radiating pain or dyspnea in the chest pain pathway), and chest pain and dizziness showed among the highest sensitivities (97.6% and 97.4%). However, a patient-facing questionnaire cannot substitute for immediate professional recognition of a stroke or acute coronary syndrome, and any deployment must ensure that use of the application never delays first contact with a triage clinician for such patients.

A methodological consideration is that the comparator was professional MTS assessment by emergency medicine clinicians rather than formal nurse-led triage. This approach was chosen to ensure that the comparator reflected a structured clinical assessment based on MTS flowcharts while maintaining blinding to the application output. The use of clinicians experienced in emergency care may have strengthened clinical judgment; however, it may also limit direct comparability with EDs where MTS is routinely performed by trained triage nurses. Therefore, future studies should validate the application against nurse-led MTS triage and assess inter-rater reliability among professional triage providers.

Although the low undertriage rate and high sensitivity are reassuring from a triage-prioritization perspective, they should not be interpreted as direct evidence of clinical safety. The present study used professional MTS assessment as the comparator and did not evaluate downstream clinical outcomes such as time to physician assessment, treatment delay, hospital admission, intensive care admission, adverse events, or return ED visits. Therefore, the findings support the application as a conservative prioritization adjunct, but not as an independently validated safety intervention.

### 4.4. Complaint-Specific Performance

Complaint category substantially influenced performance. Overtriage exceeded 70% for headache and low back pain, while urinary tract problems, diarrhea and vomiting, and eye problems showed higher concordance. This likely reflects the structure of MTS-based pathways. Complaints with potentially life-threatening differential diagnoses contain cautious discriminators that intentionally increase urgency, and subjective symptom intensity may further amplify this effect. Conversely, urinary and gastrointestinal presentations may follow more predictable symptom patterns, allowing closer alignment between patient-reported inputs and professional assessment. These findings identify specific algorithmic targets for recalibration, especially in headache and musculoskeletal pain.

The deterministic rule-based design of the application improves transparency and clinical interpretability but may also contribute to systematic overtriage. Because the algorithms were aligned with MTS urgency hierarchies, the presence of a single high-acuity patient-reported discriminator could increase the final category even without clinician verification. This mechanism is particularly relevant for headache and low back pain, in which subjective symptom severity and cautious high-risk discriminators may have driven overtriage. Future versions should preserve the hierarchical safety logic of MTS while recalibrating patient-facing wording, pain-severity thresholds, and follow-up questions for complaints with low specificity.

### 4.5. Demographic Considerations

Age showed a bidirectional relationship with discordance. Younger patients were more frequently overtriaged, whereas older patients were more frequently undertriaged. This pattern is clinically relevant because older adults often present atypically, may underreport symptoms, and are more vulnerable to adverse outcomes when assigned a lower priority. Scherer et al. similarly reported that younger patients tend to perceive their own urgency as higher than older patients do [32]. Sex and educational level were not independent predictors, suggesting that the application performed similarly across these subgroups.

### 4.6. Pain Severity

Pain scores were substantially higher among overtriaged patients. This finding is expected because pain intensity was incorporated into the MTS-aligned algorithms. However, it also illustrates a core limitation of self-assessment: subjective severity can drive urgency recommendations upward even when professional evaluation indicates lower acuity. Egoda Kapuralalage et al. reported a related finding, showing that high health anxiety was associated with self-triage decisions that did not align with professional assessment [33], and the discrepancy between patients’ perceived urgency and professional assessment is a long-recognized phenomenon [34]. Beyond health anxiety and pain intensity, several additional mechanisms of self-triage discordance have been described in the literature. Overtriage has been attributed to the deliberately risk-averse design of digital symptom assessors [18,19,28,29], to cautious high-acuity discriminators embedded in complaint pathways with potentially life-threatening differential diagnoses [21], and to the tendency of younger patients to perceive their own urgency as higher than professional assessment does [32]. Conversely, undertriage has been linked to atypical or underreported symptoms in older adults [32], to limited symptom coverage and difficulty mapping subjective complaints onto predefined categories [22,30], and to misinterpretation of question wording associated with health and digital literacy [20,27]. The complaint-specific pattern observed in the present study—overtriage concentrated in pain-dominant presentations and undertriage more frequent among older patients—is consistent with these mechanisms.

Future algorithm versions should therefore recalibrate the role of the 0–10 numerical rating scale so that pain intensity functions as a modifier rather than an isolated determinant of high urgency. For example, isolated severe pain could be prevented from automatically assigning the highest available urgency category unless accompanied by additional red-flag features, such as sudden onset, neurological deficit, systemic symptoms, cardiorespiratory symptoms, functional impairment, or other complaint-specific high-risk discriminators. Additional confirmation questions, complaint-specific pain thresholds, and age-sensitive safeguards could also be introduced. Any recalibration should be prospectively tested to ensure that specificity improves without increasing MTS-defined undertriage.

### 4.7. Strengths and Limitations

This study has several strengths. It was prospectively conducted in a real-world emergency department setting, included a large sample of consecutively enrolled adult patients, and used blinded professional Manchester Triage System assessment as the prespecified comparator. The application was locally developed and structured around a recognized triage framework, which enhances its clinical interpretability and practical relevance for emergency care settings with high patient volumes. The contribution of this study lies not in algorithmic novelty—the application is intentionally a transparent, deterministic, rule-based tool rather than an artificial-intelligence model—but in its prospective, consecutive, blinded validation against structured professional MTS assessment in a real-world, high-volume ED of a middle-income country, a setting in which patient-facing, MTS-aligned self-triage has rarely been evaluated.

Several limitations should also be acknowledged. First, the single-center design limits external validity, and the findings may not be generalizable to emergency departments with different patient populations, triage workflows, staffing models, or digital health infrastructure. The cohort was restricted to ambulatory, hemodynamically stable adults who were initially categorized as yellow or green according to the institutional three-level triage system; therefore, the application was not evaluated in critically ill patients, patients arriving by ambulance, trauma patients, or individuals with unstable vital signs. As a result, the findings should not be extrapolated to high-acuity emergency presentations. This restriction deserves emphasis. By design, the analytical cohort consisted exclusively of walk-in patients who had already been screened as hemodynamically stable and who did not carry a red (Category 1) code; the professional MTS distribution of the cohort was therefore truncated at the high-acuity end, with no Category 1 patients and only 13.6% Category 2 patients. Consequently, the sensitivity of 93.9% describes the ability of the application to recognize Categories 2–3 among a low-to-intermediate-acuity population, not its ability to detect the most time-critical presentations, which were never exposed to the tool. The application must therefore be regarded as a tool for prioritization within the walk-in stream after conventional safety screening, and its performance in unscreened, undifferentiated ED populations remains unknown.

Second, the exclusion of 970 patients (29.9% of initial registrations) who selected “Other” is an important limitation and may have introduced selection bias. This exclusion indicates that the application, in its current form, was applicable only to a predefined subset of walk-in ED presentations rather than to the full spectrum of adult walk-in patients. Therefore, the reported agreement and classification performance metrics, including the overall accuracy of 64.2%, should be interpreted as applying to patients who could identify one of the 11 available complaint categories, not to the general ED walk-in population. The descriptive comparison reported in Section 3.1 suggests that the “Other” group differed from the analytical cohort mainly in complaint type rather than in demographic profile: excluded patients were similar in sex and educational level, only slightly older, and predominantly carried musculoskeletal or extremity diagnoses that had no corresponding complaint pathway. This finding argues against a major contribution of digital or health literacy to the “Other” selection but confirms that complaint coverage was the principal driver of exclusion. Consequently, the findings may overestimate the performance of the application in routine implementation, where broader symptom coverage and more heterogeneous user characteristics would be expected. Future versions should expand complaint coverage, include clearer plain-language complaint labels, and undergo usability testing across different educational and digital-literacy groups.

Third, although professional MTS assessment was performed by emergency medicine specialists or residents who were blinded to the application output, this approach may not fully reproduce routine nurse-led triage practice. In many EDs, MTS is applied by trained triage nurses under time pressure and within specific workflow constraints, whereas physician assessment may involve different clinical judgment, risk perception, and interpretation of symptom discriminators. The use of emergency clinicians as the comparator may therefore have strengthened clinical adjudication but may also limit direct comparability with settings where MTS is routinely performed by triage nurses. In addition, the number, training level, clinical experience, and degree of MTS standardization among assessors may influence triage categorization. Moreover, each patient underwent a single professional MTS assessment; no duplicate triage assessment, independent adjudication, or formal inter-rater reliability testing was performed. This comparator therefore reflects agreement with an individual clinician’s structured MTS assignment rather than a consensus-based gold standard. Future multicenter studies should compare self-triage outputs with standardized nurse-led MTS assessment and include inter-rater reliability testing among professional triage providers.

Fourth, although the algorithms were structured according to selected MTS-related urgency logic and reviewed by emergency medicine clinicians, formal usability testing, health literacy assessment, and external algorithm validation were not performed before the present study. Therefore, some discordance may reflect not only clinical differences between patient self-report and professional assessment but also patient interpretation of the questions, digital literacy, interface-related factors, or limitations in the wording of patient-facing symptom questions. In particular, the default midpoint position of the pain slider is an interface feature that may have inflated pain-driven overtriage, and the loss of item-level response logs for 13.8% of patients limited the analysis of individual question responses. Because the application was developed as a research prototype, broader implementation would also require institutional governance, legal review, and clarification of any licensing or authorization requirements related to MTS-based digital deployment.

Fifth, the regression models had modest discrimination (AUC ≈ 0.70) and, for undertriage, a limited number of events relative to model parameters (145 events; 8.5 events per parameter), so estimates for individual complaint categories in the undertriage model should be interpreted with caution.

Finally, downstream clinical and operational outcomes were not assessed. The study therefore cannot determine whether use of the application improves patient safety, reduces waiting times, shortens emergency department length of stay, accelerates physician assessment, affects admission decisions, or reduces adverse-event rates. The observed high sensitivity and low MTS-defined undertriage rate should be interpreted as evidence of a conservative classification profile, not as direct evidence of independent clinical safety or operational effectiveness. Future studies should include clinical outcome measures and implementation endpoints to determine the real-world value of mobile self-triage in emergency department patient-flow management.

### 4.8. Future Directions

Future studies should evaluate the application in multicenter cohorts with broader complaint coverage and diverse patient populations. Algorithm refinement should focus on complaint categories with the highest overtriage, especially headache and low back pain, and on safeguards for older patients who may be more vulnerable to undertriage. Incorporating objective parameters, including vital signs or wearable sensor data, may improve specificity without compromising sensitivity. Operational studies should also examine whether self-triage reduces pre-triage waiting time, improves patient flow, or optimizes staff workload.

## 5. Conclusions

A locally developed mobile self-triage application based on MTS-related urgency logic demonstrated high sensitivity and a low MTS-defined undertriage rate for identifying urgent adult ED patients. However, exact agreement with professional MTS was modest, and frequent overtriage with low specificity limits its use as a stand-alone triage tool. These findings suggest that the application may be useful as a supervised adjunct for early prioritization and patient-flow support, but they do not establish independent clinical safety or operational effectiveness. Moreover, the high overtriage rate may increase the apparent urgent caseload and could contribute to resource misallocation if the tool is implemented without clinician oversight. Future validation should therefore evaluate not only classification performance but also operational outcomes, including pre-triage waiting time, time to clinician assessment, ED length of stay, staffing workload, resource use, and adverse-event rates. Multicenter validation, expanded symptom coverage, assessment of clinical outcomes, and targeted recalibration of high-overtriage complaint pathways are required before broader implementation.

## Figures and Tables

**Table 1:** Demographic and clinical characteristics of the study population (*N* = 2274).

Variable	Category	*n* (%)
Age, years	Mean ± SD (range)	44.4 ± 18.2 (18–94)
Sex	Female	1250 (55.0)
	Male	1024 (45.0)
Educational level	Primary school	788 (34.7)
	Middle school	320 (14.1)
	High school	547 (24.1)
	University	619 (27.2)
Time of presentation	08:00–17:00	1314 (57.8)
	17:00–24:00	836 (36.8)
	00:00–08:00	124 (5.5)
Presenting complaint	Abdominal pain	446 (19.6)
	Sore throat	321 (14.1)
	Headache	272 (12.0)
	Low back pain	226 (9.9)
	Diarrhea and vomiting	224 (9.9)
	Chest pain	183 (8.0)
	Urinary tract problems	149 (6.6)
	Dizziness	145 (6.4)
	Eye problems	116 (5.1)
	Allergic symptoms	108 (4.7)
	Shortness of breath	84 (3.7)

SD: standard deviation.

**Table 2:** Distribution of triage categories according to professional MTS and self-triage (*N* = 2274).

MTS Category	Professional MTS, *n* (%)	Self-Triage, *n* (%)
Category 2 (very urgent)	309 (13.6)	984 (43.3)
Category 3 (urgent)	929 (40.9)	918 (40.4)
Category 4 (standard)	988 (43.4)	311 (13.7)
Category 5 (non-urgent)	48 (2.1)	61 (2.7)
Total	2274 (100.0)	2274 (100.0)
Urgent (Categories 2–3)	1238 (54.4)	1902 (83.6)
Non-urgent (Categories 4–5)	1036 (45.6)	372 (16.4)

MTS: Manchester Triage System.

**Table 3:** Cross-classification matrix of self-triage and professional MTS categories (*N* = 2274).

Self-Triage/Professional MTS	MTS 2	MTS 3	MTS 4	MTS 5	Total
Self-triage 2	**254**	411	312	7	984
Self-triage 3	39	**459**	403	17	918
Self-triage 4	11	47	**242**	11	311
Self-triage 5	5	12	31	**13**	61
Total	309	929	988	48	2274

Bold diagonal values represent exact agreement. MTS: Manchester Triage System.

**Table 4:** Classification performance of self-triage in identifying MTS-defined urgent patients.

Measure	Value, %	95% CI	Numerator/Denominator
Sensitivity	93.9	92.5–95.2	1163/1238
Specificity	28.7	25.9–31.5	297/1036
Positive predictive value	61.1	58.9–63.3	1163/1902
Negative predictive value	79.8	75.4–83.8	297/372
Overall accuracy	64.2	62.2–66.2	1460/2274
Cohen’s kappa, unweighted	0.198	0.173–0.223	—
Quadratically weighted kappa	0.278	0.246–0.310	—

Professional Manchester Triage System (MTS) assessment was used as the prespecified comparator. Urgent was defined as MTS Categories 2–3 and non-urgent as Categories 4–5. Based on this threshold, true positives = 1163, false positives = 739, false negatives = 75, and true negatives = 297. Confidence intervals for classification performance measures were calculated using the exact binomial method; confidence intervals for kappa statistics were calculated using asymptotic standard errors. CI: confidence interval.

**Table 5:** Triage outcomes according to presenting complaint (*N* = 2274).

Presenting Complaint	Overtriage, %	Exact Agreement, %	Undertriage, %	*n*
Low back pain	73.0	24.3	2.7	226
Headache	72.1	25.0	2.9	272
Sore throat	62.0	34.9	3.1	321
Allergic symptoms	61.1	26.9	12.0	108
Dizziness	54.5	42.1	3.4	145
Abdominal pain	47.3	47.1	5.6	446
Chest pain	44.3	51.4	4.4	183
Shortness of breath	40.5	50.0	9.5	84
Eye problems	31.0	56.9	12.1	116
Diarrhea and vomiting	25.4	58.5	16.1	224
Urinary tract problems	24.8	67.1	8.1	149
Total	51.1	42.6	6.3	2274

Values are row percentages within each complaint group.

**Table 6:** Complaint-specific classification performance of the application in identifying professional MTS-defined urgent patients (Categories 2–3).

Presenting Complaint	*n*	MTS-Urgent, %	Sensitivity, % (95% CI)	Specificity, % (95% CI)	PPV, % (95% CI)	NPV, % (95% CI)
Abdominal pain	446	78.0	95.7 (93.0–97.4)	27.6 (19.7–37.1)	82.4 (78.4–85.8)	64.3 (49.2–77.0)
Sore throat	321	16.5	92.5 (82.1–97.0)	32.8 (27.5–38.7)	21.4 (16.6–27.2)	95.7 (89.3–98.3)
Headache	272	44.9	98.4 (94.2–99.5)	8.7 (5.1–14.3)	46.7 (40.7–52.8)	86.7 (62.1–96.3)
Low back pain	226	32.7	98.6 (92.7–99.8)	11.2 (7.1–17.2)	35.1 (28.9–41.8)	94.4 (74.2–99.0)
Diarrhea and vomiting	224	59.8	82.1 (74.7–87.7)	47.8 (37.8–58.0)	70.1 (62.5–76.7)	64.2 (52.2–74.6)
Chest pain	183	90.2	97.6 (93.9–99.1)	11.1 (3.1–32.8)	91.0 (85.8–94.4)	33.3 (9.7–70.0)
Urinary tract problems	149	65.8	94.9 (88.6–97.8)	29.4 (18.7–43.0)	72.1 (63.8–79.1)	75.0 (53.1–88.8)
Dizziness	145	80.0	97.4 (92.7–99.1)	3.4 (0.6–17.2)	80.1 (72.8–85.9)	25.0 (4.6–69.9)
Eye problems	116	31.9	81.1 (65.8–90.5)	69.6 (58.8–78.7)	55.6 (42.4–68.0)	88.7 (78.5–94.4)
Allergic symptoms	108	18.5	75.0 (53.1–88.8)	37.5 (28.1–47.9)	21.4 (13.4–32.4)	86.8 (72.7–94.2)
Shortness of breath	84	84.5	93.0 (84.6–97.0)	23.1 (8.2–50.3)	86.8 (77.4–92.7)	37.5 (13.7–69.4)
**Total**	**2274**	**54.4**	**93.9 (92.5–95.1)**	**28.7 (26.0–31.5)**	**61.1 (58.9–63.3)**	**79.8 (75.5–83.6)**

MTS-urgent, %: proportion of patients in each complaint group assigned to Category 2 or 3 by professional MTS. CI: confidence interval (Wilson method); NPV: negative predictive value; PPV: positive predictive value. Bold indicates overall values for the total cohort.

**Table 7:** Multivariable logistic regression results for overtriage and undertriage.

Variable	Overtriage aOR (95% CI)	*p* Value	Undertriage aOR (95% CI)	*p* Value
Age, per year	0.987 (0.980–0.993)	<0.001	1.015 (1.004–1.028)	0.011
Female sex	1.00 (0.83–1.19)	0.965	1.01 (0.71–1.44)	0.948
Educational level	Overall	0.215	Overall	0.441
Time of presentation	Overall	0.064	Overall	0.321
Complaint category	Overall	<0.001	Overall	<0.001
Abdominal pain	0.59 (0.38–0.90)	0.015	0.43 (0.21–0.88)	0.021
Chest pain	0.54 (0.33–0.88)	0.013	0.32 (0.13–0.80)	0.015
Diarrhea and vomiting	0.22 (0.13–0.36)	<0.001	1.37 (0.69–2.71)	0.370
Dizziness	0.83 (0.50–1.39)	0.483	0.24 (0.08–0.69)	0.008
Eye problems	0.30 (0.17–0.53)	<0.001	0.97 (0.43–2.19)	0.941
Headache	1.69 (1.05–2.71)	0.030	0.22 (0.09–0.55)	0.001
Low back pain	1.82 (1.11–2.97)	0.017	0.19 (0.07–0.52)	0.001
Shortness of breath	0.50 (0.28–0.90)	0.020	0.66 (0.26–1.70)	0.387
Sore throat	1.01 (0.64–1.59)	0.961	0.25 (0.11–0.59)	0.002
Urinary tract problems	0.23 (0.13–0.40)	<0.001	0.57 (0.25–1.32)	0.192
AUC (95% CI)	0.695 (0.674–0.717)	—	0.702 (0.658–0.745)	—
Hosmer–Lemeshow test, *p*	0.39	—	0.19	—
Nagelkerke R^2^	0.15	—	0.08	—

aOR: adjusted odds ratio; AUC: area under the receiver operating characteristic curve; CI: confidence interval; —: not applicable. Complaint categories are compared with allergic symptoms as the reference category. Both models included age, sex, educational level (four categories), time of presentation (three categories), and complaint category (11 categories); all variance inflation factors were < 5.

**Table 8:** Pain-severity scores according to triage outcome among patients reporting pain (*N* = 411).

Triage Outcome	*n*	Pain Score, Mean ± SD	*p* Value
Overtriage	219	6.9 ± 1.8	
Exact agreement	155	4.6 ± 2.2	<0.001
Undertriage	37	2.4 ± 2.2	
Total	411	5.6 ± 2.5	

Pain severity was measured using a 0–10 numerical rating scale. The *p* value was derived from one-way ANOVA; all pairwise comparisons were statistically significant (*p* < 0.001). SD: standard deviation.

## Data Availability

The datasets generated and/or analyzed during the current study are available from the corresponding author on reasonable request, subject to institutional and ethical approval.

## Supplementary Materials

The following supporting information can be downloaded at: https://www.mdpi.com/article/10.3390/jcm15176702/s1, Table S1: Demographic comparison between patients included in the analytical cohort and patients excluded because their presenting complaint was categorized as “Other”; Table S2: Distribution of discharge ICD-10 diagnoses by chapter among patients excluded because their presenting complaint was categorized as “Other”; Supplementary Material S3: Complete question sets and category assignment rules of the 11 complaint pathways of the self-triage application; Table S3.1. Abdominal Pain pathway; Table S3.2. Allergic symptoms pathway; Table S3.3. Chest Pain pathway; Table S3.4. Diarrhea and Vomiting pathway; Table S3.5. Dizziness pathway; Table S3.6. Eye Problems pathway; Table S3.7. Headache pathway; Table S3.8. Low Back Pain pathway; Table S3.9. Shortness of Breath (Dyspnea) pathway; Table S3.10. Sore Throat pathway; Table S3.11. Urinary Tract Problems pathway.
