# Supplementary material for: Agreement and Classification Performance of a Mobile Self-Triage Application Based on the Manchester Triage System in Hemodynamically Stable Adult Walk-In Emergency Department Patients: A Prospective Observational Study
Source: J Clin Med. 2026 Aug 29;15(17):6702. doi: 10.3390/jcm15176702 (PMC13566270; doi:10.3390/jcm15176702)
Supplement: Supplementary file 1 [file jcm-15-06702-s001.zip › jcm-4457845-supplementary.pdf]

Supplementary Materials

Agreement and Classification Performance of a Mobile Self-Triage Application Based on the Manchester Triage System in Hemodynamically Stable Adult Walk-In Emergency Department Patients: A Prospective Observational Study

Önler AB, Serin S, Caglar B.

Table S1. Demographic comparison between patients included in the analytical cohort and patients excluded because their presenting complaint was categorized as "Other" (N = 3,244 initial registrations).

Variable	Included (n = 2,274)	Excluded, "Other" (n = 970)	p Value
Age, years, mean ± SD	44.4 ± 18.2	47.1 ± 18.7	<0.001
Female sex, n (%)	1,250 (55.0)	517 (53.3)	0.403
Male sex, n (%)	1,024 (45.0)	453 (46.7)	
Educational level, n (%)			0.177
Primary school	788 (34.7)	379 (39.1)	
Middle school	320 (14.1)	138 (14.2)	
High school	547 (24.1)	230 (23.7)	
University	619 (27.2)	223 (23.0)	
Professional MTS category recorded, n (%)	2,274 (100.0)	57 (5.9)	N/A [a]

SD: standard deviation; MTS: Manchester Triage System; N/A: not applicable. Age was compared with the independent-samples t test; sex and educational level with the chi-square test. [a] Professional MTS categories were recorded for only 57 excluded patients (Category 2, n = 10; Category 3, n = 13; Category 4, n = 26; Category 5, n = 8), precluding agreement analysis in this group.

Table S2. Distribution of discharge ICD-10 diagnoses by chapter among patients excluded because their presenting complaint was categorized as "Other" (n = 943 with an available diagnosis code out of 970).

ICD-10 chapter	n	%
Diseases of the musculoskeletal system and connective tissue (M00–M99)	320	33.9
Diseases of the circulatory system (I00–I99)	83	8.8
Symptoms, signs and abnormal findings, not elsewhere classified (R00–R99)	66	7.0
Diseases of the digestive system (K00–K93)	62	6.6
Diseases of the respiratory system (J00–J99)	61	6.5
Factors influencing health status and contact with health services (Z00–Z99)	60	6.4
Diseases of the eye and adnexa; ear and mastoid process (H00–H95)	56	5.9
Diseases of the skin and subcutaneous tissue (L00–L99)	56	5.9
Diseases of the genitourinary system (N00–N99)	52	5.5
Mental and behavioural disorders (F00–F99)	30	3.2
Certain infectious and parasitic diseases (A00–B99)	23	2.4
Injury, poisoning and certain other consequences of external causes (S00–T98)	18	1.9
Endocrine, nutritional and metabolic diseases (E00–E90)	17	1.8
Diseases of the nervous system (G00–G99)	14	1.5
External causes of morbidity (V01–Y98)	12	1.3
Diseases of the blood (D50–D89)	8	0.8

ICD-10 chapter	n	%
Pregnancy, childbirth and the puerperium (O00–O99)	5	0.5
**Total**	**943**	**100.0**

ICD-10: International Statistical Classification of Diseases and Related Health Problems, 10th Revision. Within the musculoskeletal chapter, 301 of 320 codes (94.1%) were M79 (other soft-tissue disorders, predominantly limb pain); within the symptom chapter, the most frequent code was R53 (malaise and fatigue; n = 16). A diagnosis code was unavailable for 27 patients (2.8%).

Supplementary Material S3. Complete question sets and category assignment rules of the 11 complaint pathways of the self-triage application.

Each pathway consisted of sequential decision boxes presented in order. All questions were answered Yes/No on the tablet. If any question in a box was answered Yes, the pathway terminated and the category shown for that box was assigned; if all questions in the box were answered No, the next box was displayed. In the abdominal pain, chest pain, and sore throat pathways, patients who answered No to all questions in Boxes 1 and 2 were shown a 0–10 pain-intensity slider whose value determined the category (8–10, Category 2; 5–7, Category 3; 1–4, Category 4; 0, Category 5). In the headache and low back pain pathways, patients who did not endorse a Box 1 or Box 2 discriminator proceeded to Box 3 and then to the pain-intensity slider; the final category was the higher-acuity of the Box 3 result and the pain-score result. The slider was initially displayed at the midpoint (5) and could be confirmed without being moved. Category 1 (immediate) was not assignable by the application. Question wording is a direct English translation of the Turkish patient-facing text.

Table S3.1. Abdominal Pain pathway.

Decision box	Patient-facing questions	Category assignment
Box 1	1. Have you vomited a large amount of blood?<br>2. Have you had bright red rectal bleeding?<br>3. Do you have a known pregnancy?<br>4. Does your pain radiate to your back?<br>5. Have you had a fever of 41°C or higher?	Any Yes → Category 2
Box 2	1. In the last 24 hours, have you seen blood mixed with your vomit?<br>2. Have you had black, foul-smelling stools?<br>3. Are you vomiting in a way that prevents you from eating or drinking?<br>4. Is there a possibility that you might be pregnant?<br>5. Have you had a fever of 38.5°C or higher?	Any Yes → Category 3
Terminal node	All questions in Boxes 1–2 answered No → pain-intensity slider	Pain score (0–10): 8–10 → Category 2; 5–7 → Category 3; 1–4 → Category 4; 0 → Category 5

Table S3.2. Allergic symptoms pathway.

Decision box	Patient-facing questions	Category assignment
Box 1	1. Have you developed swelling of your tongue or face?<br>2. Do you have difficulty speaking?<br>3. Do you have new-onset palpitations, chest pain, or shortness of breath?<br>4. Have you ever lost consciousness due to an allergic reaction before?	Any Yes → Category 2
Box 2	1. Do you have red, raised rashes covering most of your body?<br>2. Do the skin lesions cause pain?	Any Yes → Category 3
Box 3	1. Do you have redness, pain, or itching limited to the exposed area?<br>2. Did these symptoms develop within the last week?	Any Yes → Category 4
Terminal node	All questions answered No	Category 5

Table S3.3. Chest Pain pathway.

Decision box	Patient-facing questions	Category assignment
Box 1	1. Do you have difficulty breathing?<br>2. Do you have palpitations together with chest pain?<br>3. Do you feel pressure or tightness in your chest?<br>4. Does the pain radiate to your neck, jaw, or arm?	Any Yes → Category 2
Box 2	1. Are you vomiting in a way that prevents you from eating or drinking?	Any Yes → Category 3

Decision box	Patient-facing questions	Category assignment
	2. Does your pain occur when you breathe or cough?<br>3. Do you have a known history of heart disease or previous coronary angiography?	
Pain-intensity slider	All questions in Boxes 1–2 answered No	Pain score (0–10): 8–10 → Category 2; 5–7 → Category 3; 1–4 → Category 4; 0 → Category 5

Table S3.4. Diarrhea and Vomiting pathway.

Decision box	Patient-facing questions	Category assignment
Box 1	1. Have you vomited blood? Does blood or black, jelly-like material appear every time you vomit?<br>2. Have you had bright red rectal bleeding?<br>3. Have you had a fever of 41°C or higher?	Any Yes → Category 2
Box 2	1. Have you had bloody vomiting within the last 24 hours?<br>2. Do you have black, foul-smelling stools?<br>3. Are you vomiting to the extent that you cannot eat or drink?<br>4. Have you had a fever of 38.5°C or higher?	Any Yes → Category 3
Box 3	1. Did these symptoms start within the last week?<br>2. Do you frequently experience similar symptoms?	Any Yes → Category 4
Terminal node	All questions answered No	Category 5

Table S3.5. Dizziness pathway.

Decision box	Patient-facing questions	Category assignment
Box 1	1. Have you experienced a head injury?<br>2. In the last 24 hours, have you developed numbness or weakness in your arms or legs, speech difficulty, or double vision?<br>3. Have you had chest pain or palpitations associated with dizziness?<br>4. Have you had a severe headache together with dizziness?	Any Yes → Category 2
Box 2	1. Have you had nausea or vomiting?<br>2. Does your dizziness occur repeatedly?<br>3. Do you have difficulty walking or loss of balance?<br>4. Do you have hypertension or diabetes?	Any Yes → Category 3
Box 3	1. Does your dizziness occur when you move your head?<br>2. Have you experienced similar dizziness before?<br>3. Did your symptoms start within the last week?	Any Yes → Category 4
Terminal node	All questions answered No	Category 5

Table S3.6. Eye Problems pathway.

Decision box	Patient-facing questions	Category assignment
Box 1	1. Have you experienced vision loss within the last 24 hours?<br>2. Has a sharp object penetrated your eye?	Any Yes → Category 2
Box 2	1. Have you experienced visual disturbance within the last week?	Any Yes → Category 3
Box 3	1. Do you have redness in your eye?<br>2. Has a foreign body entered your eye?<br>3. Do you have double vision?<br>4. Did these symptoms start within the last week?	Any Yes → Category 4

Decision box	Patient-facing questions	Category assignment
Terminal node	All questions answered No	Category 5

Table S3.7. Headache pathway.

Decision box	Patient-facing questions	Category assignment
Box 1	1. Did your headache start suddenly within the last hour?<br>2. In the last 24 hours, have you developed numbness or weakness in your arms or legs, urinary or fecal incontinence?<br>3. Do you have a newly developed red rash on your skin?<br>4. Do you have decreased vision or vision loss associated with your headache?<br>5. Have you had a fever of 41°C or higher?	Any Yes → Category 2
Box 2	1. In the last week, have you experienced decreased vision or vision loss?<br>2. Have you lost consciousness?<br>3. Are you vomiting in a way that prevents you from eating or drinking?<br>4. Have you had a fever of 38.5°C or higher?	Any Yes → Category 3
Box 3	1. Do you have nausea or vomiting?<br>2. Have you had similar headaches within the last week?<br>3. Do you have a fever below 38.5°C?	Any Yes → Category 4
	All questions answered No	Category 5
Pain-intensity slider	Displayed after Box 3	Pain score (0–10): 8–10 → Category 2; 5–7 → Category 3; 1–4 → Category 4; 0 → Category 5

Table S3.8. Low Back Pain pathway.

Decision box	Patient-facing questions	Category assignment
Box 1	1. In the last 24 hours, have you experienced numbness or weakness in your arms or legs?<br>2. Have you developed new urinary incontinence or fecal incontinence associated with your back pain?<br>3. Have you fallen from a height?<br>4. Do you have abdominal pain accompanying your back pain?<br>5. Have you had a fever of 41°C or higher?	Any Yes → Category 2
Box 2	1. Have you hit your back/waist against something or experienced trauma?<br>2. Do you have difficulty walking?<br>3. Is your pain increasing in severity or recurring continuously?<br>4. Have you had a fever of 38.5°C or higher?	Any Yes → Category 3
Box 3	1. Did these symptoms start within the last week?<br>2. Do you have a known history of low back disease?	Any Yes → Category 4
	All questions answered No	Category 5
Pain-intensity slider	Displayed after Box 3	Pain score (0–10): 8–10 → Category 2; 5–7 → Category 3; 1–4 → Category 4; 0 → Category 5

Table S3.9. Shortness of Breath (Dyspnea) pathway.

Decision box	Patient-facing questions	Category assignment
Box 1	1. Is your shortness of breath severe enough to prevent you from speaking?<br>2. Have you experienced any loss of consciousness?<br>3. Do you have chest pain or newly developed palpitations?<br>4. Have you previously experienced an asthma or COPD attack?<br>5. Have you had a fever of 41°C or higher?	Any Yes → Category 2
Box 2	1. Do you have chest pain when breathing or coughing?<br>2. Do you have a fever of 38.5°C or higher?	Any Yes → Category 3
Box 3	1. Do you have wheezing while breathing?<br>2. Do you have a productive cough (phlegm)?<br>3. Do you have a known chronic lung disease?	Any Yes → Category 4
Terminal node	All questions answered No	Category 5

Table S3.10. Sore Throat pathway.

Decision box	Patient-facing questions	Category assignment
Box 1	1. Are you receiving chemotherapy or radiotherapy? Do you have a disease affecting your immune system?<br>2. Are you experiencing severe shortness of breath?<br>3. Have you experienced loss of consciousness or fainting?<br>4. Have you had a fever of 41°C or higher?	Any Yes → Category 2
Box 2	1. Do you have difficulty swallowing even liquids?<br>2. Have you traveled abroad within the last 2 weeks?<br>3. Do you have a fever of 38.5°C or higher?	Any Yes → Category 3
Terminal node	All questions in Boxes 1–2 answered No → pain-intensity slider	Pain score (0–10): 8–10 → Category 2; 5–7 → Category 3; 1–4 → Category 4; 0 → Category 5

Table S3.11. Urinary Tract Problems pathway.

Decision box	Patient-facing questions	Category assignment
Box 1	1. Do you have a known cancer diagnosis? Are you currently receiving chemotherapy or radiotherapy?<br>2. Have you had a fever of 41°C or higher?	Any Yes → Category 2
Box 2	1. Have you had red-colored urine or noticed blood in your urine?<br>2. Are you vomiting to the extent that you cannot eat or drink?<br>3. Do you have difficulty urinating, and does it cause pain?<br>4. Do you have flank or lower back pain that comes and goes in severity?	Any Yes → Category 3
Box 3	1. Do you have nausea or vomiting?<br>2. Do you experience burning or pain while urinating?<br>3. Did these symptoms start within the last week?	Any Yes → Category 4
Terminal node	All questions answered No	Category 5
